# Mining Skeletal Phenotype Descriptions from Scientific Literature

**DOI:** 10.1371/journal.pone.0055656

**Published:** 2013-02-08

**Authors:** Tudor Groza, Jane Hunter, Andreas Zankl

**Affiliations:** 1 School of ITEE, The University of Queensland, Australia; 2 Bone Dysplasia Research Group, UQ Centre for Clinical Research (UQCCR), The University of Queensland, Australia; 3 Genetic Health Queensland, Royal Brisbane and Women’s Hospital, Herston, Australia; UT MD Anderson Cancer Center, United States of America

## Abstract

Phenotype descriptions are important for our understanding of genetics, as they enable the computation and analysis of a varied range of issues related to the genetic and developmental bases of correlated characters. The literature contains a wealth of such phenotype descriptions, usually reported as free-text entries, similar to typical clinical summaries. In this paper, we focus on creating and making available an annotated corpus of skeletal phenotype descriptions. In addition, we present and evaluate a hybrid Machine Learning approach for mining phenotype descriptions from free text. Our hybrid approach uses an ensemble of four classifiers and experiments with several aggregation techniques. The best scoring technique achieves an F-1 score of 71.52%, which is close to the state-of-the-art in other domains, where training data exists in abundance. Finally, we discuss the influence of the features chosen for the model on the overall performance of the method.

## Introduction

Phenotype descriptions are important for our understanding of genetics, as they enable the computation and analysis of a varied range of issues related to the genetic and developmental bases of correlated characters [1]. The literature contains a wealth of such phenotype descriptions, usually reported as free-text entries, similar to typical clinical summaries (see, for example, the Online Mendelian Inheritance in Man (OMIM) knowledge base [2]). More concretely, they take the shape of statements that describe qualitative aspects (or sometimes abnormalities) of biological states, or in our case, of anatomical entities, e.g., *middle phalanges of the hand are short*. In order to take full advantage of this knowledge and to work towards enabling diverse automated and rich processing services, we first need to close the gap between their plain textual representation and a machine-processable format. More concretely, we need to perform the task of biomedical named entity recognition (Bio-NER), with a focus on phenotype descriptions. This would enable, among other services, genotype–phenotype or disorder–phenotype correlation analysis and reasoning, i.e., the analysis of associations between gene mutations and observed phenotypic characteristics – e.g., a mutation in the FGFR3 gene leads to short stature, or of associations between observed phenotypic features and disorders – e.g., short stature is a feature of Achondroplasia.

There has been a significant amount of research performed, over the course of the last few years, on modelling and formalising phenotype descriptions. For example, the Human Phenotype Ontology (HPO) project [3] has curated the most comprehensive ontology of human phenotype descriptions to-date, consisting of over 10,000 terms. Similar other projects have followed, e.g., the Mammalian Phenotype Ontology [4]– concentrated on mammalian phenotype descriptions – or the Elements of Morphology Project [5]– focusing on phenotypic variations of the head and face. Formalised phenotypic descriptions have then been successfully used for studying cross-species phenotype networks [6], [7], linking human diseases to animal models [8] or predicting diagnoses using semantic similarity measures [9], [10]. However, all of the above-mentioned projects have either curated phenotype descriptions manually, or have built tools that support the manual curation process – we are currently not aware of any attempt to extract such descriptions automatically from scientific literature.

The context of our research is provided by the SKELETOME project [11], which aims to create a community-driven knowledge curation platform for the skeletal dysplasia domain. Bone dysplasias are a group of heterogeneous genetic disorders that affect predominantly the skeletal development. Patients diagnosed with such disorders suffer from complex medical issues that can be described via clinical findings, e.g., pains in limbs, radiographic findings, e.g., bilateral arachnodactyly and genetic findings, e.g., deletion mutation in FGFR3. One of the main features of the SKELETOME platform is the semantic annotation of clinical summaries (i.e., automatic extraction of and annotation of clinical summaries with ontological entities), process that is currently limited to concepts defined by HPO. We are, hence, interested in developing mechanisms that identify skeletal phenotype descriptions independently of their presence in a particular ontology. Nevertheless, the same mechanism can also be employed to enrich or populate current phenotype ontologies, or as a pre-processing step in mining disorder-phenotype associations in domains focused on the skeletal and muscular system.

The task of biomedical named entity recognition aims to automatically analyse free text (usually in the form of scientific publications) and to extract “named entities” specific to a particular domain and goal. It is, in principle, the prerequisite step for any advance processing tasks, including ontology population (i.e., creating instances of particular concepts in a given ontology) or relation extraction (e.g., more concretely protein-protein interactions). Over the past years, the Bio-NER field has flourished, especially in areas such as gene/protein mention tagging or gene normalisation, with an impressive number of approaches being proposed. Currently, the state-of-the-art F1 scores are in the range of 86%–87% and come closer to 90% if post-processing steps are used [12].

In general Bio-NER represents a complex task due to non-standardised naming schemes, ambiguity and evolution. However, our goal of recognising phenotype descriptions adds a series of additional domain-specific challenges, as listed next: [(i)] **ambiguity**, i.e., the same term may refer to multiple different entities – for example, *irregular ossification of the proximal* **radial** *metaphisis* vs. **radial** *club hand* – *radial* refers to the anatomical entity *radius* in the former case and the anatomical coordinate *radial* in the latter, or *short* **long** *bones* vs. **long** *metacarpals* – *long* acts as part of an anatomical entity name in the former and represents a quality in the latter. This domain also suffers from a special case of semantic ambiguity in which the text requires human interpretation to judge whether it represents or not a phenotype description that is worth noting – e.g., *11 pairs of ribs* or *6 fingers*. **use of abbreviations** – for example, *segmentation defects in* **L4** *-* **S1** **use of metaphorical expressions** – e.g., **bell-shaped** *thorax*, **hitchhiker** *thumb*, **bone-in-bone** *appearance* **use of hedging and various forms of qualifiers** – e.g., **subtle** *flattening and squaring of the metacarpal heads*, *segmentation defects* **appear** *to affect L4-S1* **complex intrinsic structure** – the lexical structure of phenotype descriptions may take several forms. They may have a canonical form, i.e., a conjunction of well-defined quality-entity pairs – *bell-shaped thorax* or a non-canonical form, in which entities and qualities are associated either via verbs (e.g., *Vertebral-segmentation defects are most severe in the cervical and thoracic regions*) or via conjunctions (e.g., *short and wide ribs with metaphyseal cupping*). At the same time, each component of a phenotype description may have a nested structure, as in *flattening, underdevelopment, and squaring of the heads of the metacarpal bones, particularly at metacarpal IV bilaterally*. All these challenges, and in particular the latter three, makes the identification of the boundaries of phenotype descriptions particularly difficult.

As mentioned, the Bio-NER field consists of a wealth of algorithms and methods, which can usually be classified into three categories: dictionary-based, rule-based and statistical machine learning methods. Dictionary and rule-based approaches achieve satisfactory results, especially in the context of gene/protein mention tagging, and rely on thesauri and manually crafted rules to perform exact or partial matching. Unfortunately, such methods (on their own) are not feasible for skeletal phenotype descriptions, due to the ambiguous nature and complex intrinsic structure of such descriptions. A combined approach could be envisioned, by using manually created rules to recognise the structure of the phenotype descriptions and complement them with quality and anatomical dictionaries to spot the key concepts. However, such rules are hard to maintain and even harder to create for complex nested skeletal phenotypes.

A set of approaches that have proved to perform extremely well in Bio-NER are the machine learning (ML) methods. They are robust and versatile, and are capable to detect patterns that cannot be easily expressed in rules and concepts that are not present in dictionaries. The main drawback of ML methods is the necessity of training data (that has to contain, in principle, a fair distribution of positive and negative examples for the target classes), which in some domains exist in abundance (see gene/protein NER [13], [14]) while in others, e.g., our domain, is completely absent. As a side remark, one could use HPO, for example, as a bootstrapping dictionary in the training phase. However, this comprises only clean and mostly canonical phenotype descriptions, and thus covers only a fraction of all possible forms. HPO concepts cannot be directly used for recognition in free text because they are missing the surrounding context. Although the process of creating a training corpus is cumbersome, and many see it as complex as creating and maintaing rules in a rule-based approach, current ML algorithms provide a much better value than any other methods, even for smaller sized training corpora.

Most of the existing Bio-NER ML approaches create and use models for a particular technique, such as, Support Vector Machines (SVM) [15], Conditional Random Fields (CRF) [16], Hidden Markov Model (HMM) or Maximum Entropy (MaxEnt) (e.g., [17], [18], [19]). Lately, the research has shifted towards combining several such methods into ensembles of classifiers, with the goal of achieving higher performances [20]. Continuing this trend, we propose an ensemble of four classifiers (two CRF and two SVM), in addition to experimenting with multiple aggregation strategies, such as simple and paired set operations or simple voting mechanism with veto.

This paper brings two general contributions to the phenotype modelling and analysis domain: [(i)] we introduce and make available an annotated corpus of skeletal phenotype descriptions – to be used by researchers to advance Bio-NER methods in this area, and we propose a first hybrid method for mining phenotype descriptions from free text. As we will show, our approach achieves very promising results, 71.52% F-1 score, comparable with the state-of-the-art in other biomedical domains, where training data does not represent an issue.

Finally, for clarification purposes, we need to underline that the focus of our research described in this manuscript is the recognition of phenotype descriptions in free text. Subject to the specific goal, this step can then be followed by segmentation, if one is interested in capturing entity-quality statements, or alignment, if the goal is mapping to ontological concepts. As such, each of these aspects represent research topics on their own and are, thus, out of the scope of this manuscript.

## Materials and Methods

### Data

In order to train and test the classifiers used for recognising phenotype descriptions, we have manually compiled a corpus of figure captions from 395 random publications from the Springer Paediatric Radiology Journal, the American Journal of Human Genetics and the American Journal of Medical Genetics Part A. We chose to use figure captions because: (i) they are easier to collect and compile into a corpus, and (ii) phenotype descriptions present within them follow the same structure and format as in clinical summaries or main bodies of scientific publications. As a remark, all figure captions are represented by plain text and do not have a particular structure or format, except for the figure number, which is not necessarily always present.

Statistics on the corpus are available in Table 1. It consists of 1,194 figure captions that capture a total of 5,423 phenotype descriptions. On average, a figure caption has 53 tokens, i.e., around three sentences, and contains four phenotype descriptions. Phenotype descriptions have, in average, five tokens per entry, with the size ranging from one to 31. The corpus is available at: http://purl.org/skeletome/corpora/pheno_corpus.

**Table 1:** Statistics of the phenotype descriptions corpus.

Total number of figure captions	1,194
Total number of tokens	64,052
Average number of tokens per caption	53
Total number of phenotype descriptions	5,423
Average number of phenotype descriptions per caption	4
Average number of tokens per phenotype description	5
Maximum number of tokens in one phenotype description	31
Minimum number of tokens in one phenotype description	1

The corpus used for training the classifiers has been manually compiled from 395 random publications from three different academic journals. It consists of 1,194 image captions that describe 5,423 phenotype descriptions. The total number of tokens in the corpus is 64,052, with an average of 5 tokens per phenotype description. The longest phenotype description comprises 31 tokens, while the shortest consists of only one token.

Composite set aggregations achieve a lower performance than direct set operations, due to the use of the entire ensemble of classifiers. The best result is, nevertheless, fairly close to the best individual classifier performance. An interesting aspect is the corrective role carried by MALLET in intersection settings. The highest precision (70.34% and 70.02%) is achieved by combining the intersection of MALLET with CRF++ (which has the lowest individual results –35% less precision than MALLET) with the intersection of the two YamCha classifiers, which behave fairly similar.

The annotation of the phenotype descriptions has been performed by a clinical geneticist, expert in bone dysplasias, using the DOMEO [21] annotation platform. DOMEO provides a versatile environment to collaboratively create and share discourse or domain-driven stand-off annotations. No particular annotation guidelines have been created for this task since, as mentioned, the textual representation of phenotype descriptions in figure captions is the same as in clinical reports or case studies listed in publications, and the marking has been performed by an expert. Also, from an expert interpretation perspective – similar to gene or protein mentions – there are, in principle, no ambiguity issues. The only two aspects that have been specified in the context of the actual annotation process were the following: (i) leading articles should be left out since they don’t provide any insights into the semantics of the phenotype descriptions, and (ii) phenotype descriptions, in particular the ones taking a non-canonical form, should be complete, in order to enable further processing of their semantics – e.g., including the verb that connects an anatomical entity to a quality or that confirms or negates the phenotype description. This last aspect is crucial for a correct automatic segmentation, and later interpretation, of the phenotype descriptions, and it is the one that makes the recognition process particularly difficult.

### Methods

Our method relies on an ensemble of four classifiers, trained and tested on the above-described corpus. For training purposes, phenotype descriptions within the corpus have been labelled according to the *BIO* scheme, i.e., **B** – beginning of a phenotype description; **I** – inside a phenotype description; **O** – outside a phenotype description. Tests have been performed by training individually each classifier via a ten-fold cross-validation with stratification.

#### Classifiers

We have used the following packages to build the four divergent classifiers:

- two Conditional Random Fields (CRF) chunkers: MALLET [22] and CRF++ (http://crfpp.googlecode.com/). Both packages are freely available and were used to train forward parsing chunkers. MALLET has been trained without feature induction and with the heuristic option for weights selection (some-dense), while the CRF++ chunker has been trained with the hyperparameter set to 3.5.
- two Support Vector Machines-based chunkers provided by the YamCha package [23], both using multi-class classifiers trained with a second degree polynomial kernel. The difference between the two was the training method: one was trained using the one vs. one method, while the other using the one vs. all method.

#### Aggregation strategies

In addition to individual chunking/classification, we have also experimented with two different aggregation schemes:

- set operations – results of the individual classifiers have been treated as sets, which have then been combined using direct or aggregated operations. Direct operations refer to union and intersections between pairs of classification results (e.g., CFR++ 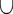 MALLET), while aggregated operations refer to combinations of unions and/or intersections of paired classification results (e.g., (CRF++ 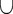 MALLET) 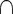 (YamCha1vs1 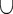 YamCha1vsAll)).
- simple majority voting with veto – the winning result within a simple majority voting is the one that receives 50% or more votes. Since we have an even number of classifiers, we’ve introduced the veto option, i.e., in the case of a tie or of a complete disagreement, the winning result is provided by the veto owner.

#### Features for classification

We used four types of features to build classification models, detailed in the rest of this section.

*Simple features* consider the basic elements of a token, such as, the prefix, suffix, lemma and part of speech. There are six particular features we have used:

- the prefix of the token, i.e., the first n adjacent characters of the token, for a variable n. For example, for n = 5 and the “flattening” token, the prefix would be: *f fl fla flat flatt*
- the suffix of the token - similarly to the prefix, but considering the last n adjacent characters. E.g., *g ng ing ning ening* – for n = 5 and the token “flattening”.
- two lemma features, one provided by a typical shallow natural language processing framework (we’ve used GATE [24]) and one provided by SPECIALIST Lexicon [25]
- two part of speech tag features, again, one provided by the same shallow NLP toolkit, and one by the SPECIALIST Lexicon.

*Morphological features* consider the internal structure of the token. Here, we were interested in the presence of digits, vowels and punctuation. Five features have been used:

- punctuation – signals the presence of punctuation characters in the token, e.g., comma, period, etc.
- vowels – builds the shape of the token by replacing all consonants with an arbitrary character. For example, “flattening” is represented by –a–e-i–.
- digits – all digits in the token are replaced by an arbitrary character (e.g., ‘*’). If no digits are present, the entire token is replaced by a standard one, e.g., no*
- the shape of the token – formed by replacing all capital letters with an arbitrary capital letter (e.g., ‘A’), all non-capital letters by an arbitrary non-capital letter (e.g., ‘a’) and all digits by an arbitrary digit (e.g. ‘0’). For example, the token “Flattening” would have the shape Aaaaaaaaaa.
- the brief shape of the token – a compressed version of the shape where all the same adjacent characters are compressed into one, e.g., for “Flattening” – Aa.

*Dictionary-based features* use external resources to signal the presence of specific elements within the tokens. We’ve experimented with six dictionaries, four generic ones and two domain specific:

- ordinals – denotes the presence of ordinals, e.g., 1st, 2nd, etc
- conjuctions – denotes the presence of conjunctions, e.g., and, or
- connectives – signals connective tokens, e.g., at, in, of, etc
- coordinates – shows the presence of coordinates, e.g., central, left, etc
- anatomy – an unigram dictionary compiled from the Foundational Model of Anatomy (FMA) [26] denoting anatomical concepts.
- quality – an unigram dictionary compiled from the Phenotype and Trait Ontology (PATO) [27] denoting qualities.

*Token contexts.* In addition to single token features, we’ve experimented also with n-gram token contexts of variable sizes. The context of a token is provided by the window of size n, centred in the current token and considering the 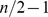 neighbouring tokens to the left and to the right. Within our experiments, we’ve used window sizes ranging from 1 to 5. Neighbouring tokens can be considered individually, thus resulting in unigram contexts, two at a time – resulting in bigram contexts, or three at a time – resulting in trigram contexts. There could be multiple other configurations, however, we’ve only used these three.

## Results and Discussion

### Experimental Results

We have performed an extensive evaluation of the ensemble of classifiers by taking into account different combinations of features, and hence different classification models. The evaluation procedure was via ten-fold cross-validation with stratification, to eliminate a possible bias. For each fold we have calculated the Precision, Recall and F-1 score, while the final evaluation metrics are represented by the average across the ten folds. In the following sections we discuss the best-achieving models within each category of aggregation techniques.

#### Individual classifiers

Table 2 lists the results achieved by the individual classifiers with and without the use of the two domain-specific dictionaries (i.e., anatomy and quality). We can see that MALLET has constantly outperformed all the other methods, with F-1 scores of 70.76% and 69.02% respectively, which demonstrates the superiority of the CRF package in tag labelling. The surprising aspect of this round of tests has been the decrease in performance of the MALLET chunker when using domain-specific dictionaries (−1.74%). The same phenomenon also appears in the case of CRF++, while the two YamCha classifiers had an opposite behaviour – behaviour which we would have considered more natural.

**Table 2:** Evaluation results for individual classifiers – with and without domain-specific dictionaries.

Method	Without dictionaries			With dictionaries		
	P (%)	R (%)	F-1 (%)	P (%)	R (%)	F-1 (%)
Mallet	76.93	65.51	**70.76**	74.91	63.99	**69.02**
CRF++	41.71	53.65	46.93	41.33	53.58	46.66
YamCha1vs1	67.48	62.38	64.83	68.43	63.36	65.80
YamCha1vsAll	68.61	62.17	65.23	68.62	62.48	65.40

We can see that MALLET constantly outperforms all the other approaches, with a margin of almost 5% without using dictionaries and almost 3% when using domain-specific dictionaries. The surprising aspect is the decrease in performance when using dictionaries as opposed to the setting that omits them.

These results have been achieved using the following combinations of features:

- MALLET: all simple and morphological features, the generic dictionaries and a single token context feature comprising of token bigrams with a window of 3;
- CRF++: all simple and morphological features, the generic dictionaries and two token context features: token bigrams with a window of 3 and token unigrams with a window of 5;
- YamCha1vs1 and YamCha1vsAll: all simple and morphological features, the generic dictionaries and a single token context feature: token unigrams with a window of 5.

#### Set operations

Two types of aggregation techniques have been used in the context of set operations: simple set operations between pairs of classifiers and composite set operations between pairs of simple set operations. In addition, two set operators have been used: union and intersection. The union of two classification results considers all outcomes to be correct and hence maintains everything. The effect of the union operator is usually visible in the increase of recall. On the other hand, intersection retains only those outcomes that are present in both classification results, hence increasing the overall precision.

Table 3 presents the results achieved via direct set operations. Naturally, the best results have been achieved by pairs that contained the MALLET chunker – the best in the individual category. The results of both unions of MALLET with the YamCha classifiers outperform the individual classification with 0.10% and 0.54% without using domain-specific dictionaries, and with 1.64% and 1.62% when using the dictionaries. The effect of the set operators can be clearly seen in these results. The union operations have increased the recall with almost 13% when compared against the individual MALLET results, at the expense of precision, which has dropped proportionally. Similarly, the intersection of MALLET with any of the YamCha classifiers has boosted the precision, reaching almost 90% without the use of domain dictionaries.

**Table 3:** Evaluation results for the simple set aggregation technique – with and without domain-specific dictionaries.

Aggregation	Without dictionaries			With dictionaries		
	P (%)	R (%)	F-1 (%)	P (%)	R (%)	F-1 (%)
Mallet 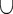 YamCha1vs1	64.61	*78.44*	70.86	64.24	*78.49*	**70.66**
Mallet 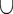 YamCha1vsAll	65.42	78.33	**71.30**	64.58	77.90	70.62
YamCha1vs1 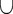 YamCha1vsAll	66.25	64.85	65.54	67.02	64.79	65.89
YamCha1vs1 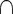 YamCha1vsAll	70.74	59.71	64.76	70.80	61.05	65.57
Mallet 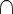 YamCha1vsAll	*89.06*	49.35	63.51	*87.91*	48.57	62.57

The best scoring direct set operations are those that include MALLET in their composition, which is in line with the individual classification results. The italicised results demonstrate the effect of the set operations: union increases the recall with almost 13%, while intersection increases the precision with around 12%.

The second category of set operations has achieved scores lower that the direct one, because of the presence of CRF++ in the aggregation. Table 4 lists a series of composite set operations. Among these, the best scoring one is the intersection between the unions of MALLET and YamCha1vAll, and CRF++ and YamCha1vs1, respectively (68.48% F-1), which came close to the best F-1 achieved individually by MALLET. An interesting result was the third one in Table 4, where we can see the highest achieved precision (with or without dictionaries) in this round of tests. Taking into consideration the results of the direct set operations, it seems that intersection of MALLET with any of the other classifiers performs extremely well, even if the initial precision of the individual classifiers is fairly low – this has been the case of CRF++ that achieved a 41.71% precision, however, when combined with MALLET, the resulting precision reached 87%. Consequently, we can can conclude here that MALLET is an extremely valuable tool for the chunking task in scenarios that require a very high precision.

**Table 4:** Evaluation results for the paired set aggregation technique – with and without domain-specific dictionaries.

Aggregation	Without dictionaries			With dictionaries		
	P (%)	R (%)	F-1 (%)	P (%)	R (%)	F-1 (%)
(Mallet 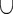 YamCha1vs1) 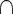 (CRF++ 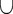 YamCha1vsAll)	70.06	66.75	68.36	69.62	67.53	68.56
(Mallet 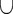 YamCha1vsAll) 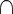 (CRF++ 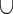 YamCha1vs1)	70.05	66.97	**68.48**	69.76	67.53	**68.62**
(Mallet 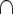 CRF++) 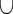 (YamCha1vs1 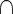 YamCha1vsAll)	*70.34*	65.91	68.06	*70.02*	66.72	68.33

#### Voting mechanism

As already mentioned, the voting mechanism used a simple majority aggregation, with a veto option in the case of a perfect tie or complete disagreement. Results of this scheme are presented in Table 5, listed according to the veto owner. Again, the best achieving classifier is the one having MALLET as the veto owner, in both categories (with or without domain-specific dictionaries). There are two remarks that are worth noting here: (i) when MALLET is the veto owner, the voting mechanism produces a reverse of scores in precision and recall – more concretely, the precision has dropped almost perfectly proportional with the increase in recall; and (ii) the increase in F-1 when compared against the individual classification results has been more remarkable when CRF++ is the veto owner, with almost 8% than in the case of the ensembles having the other classifiers as veto owner, i.e., YamCha1vs1 and YamCha1vsAll (almost 3%) and MALLET (almost 1%).

**Table 5:** Evaluation results for the voting aggregation technique – with and without domain-specific dictionaries.

Veto owner	Without dictionaries			With dictionaries		
	P (%)	R (%)	F-1 (%)	P (%)	R (%)	F-1 (%)
Mallet	66.14	77.84	**71.52**	65.29	77.95	**71.06**
CRF++	44.10	70.73	54.33	43.78	71.53	54.31
YamCha1vs1	67.42	68.79	68.10	67.52	69.21	68.35
YamCha1vsAll	68.38	68.70	68.54	68.06	68.82	68.44

The results of the voting method are in line with the rest of the aggregation methods. The highest score (71.52%/71.06%) is achieved by using MALLET as veto owner.

## Discussion

In order to get a better understanding of the role and importance of the features used for classification, we’ve performed two additional experiments with the MALLET chunker. In the first experiment we’ve trained MALLET with each individual feature from the overall best model (in addition to a couple more that were part of the models of the other classifiers), while in the second experiment, we’ve trained MALLET using a leave-one-out setting, i.e., we’ve used the best MALLET model from which we’ve left out one feature at a time. Both experiments used a ten-fold cross validation with stratification.

**Figure 1:**
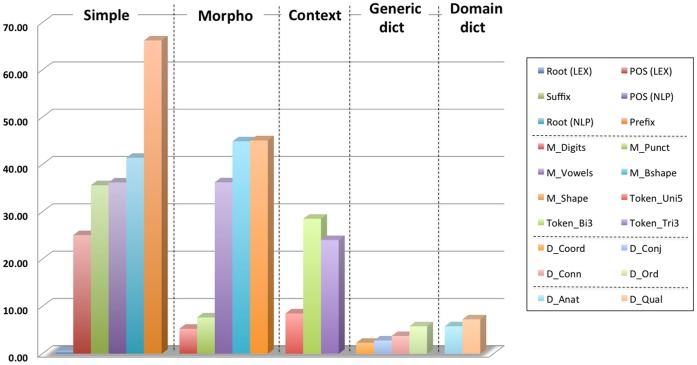
Evaluation results for MALLET ten-fold cross validation using single features. The graph groups the features according to the categories used to describe them in the Materials and Methods section. We can observe that the simple and morphological features perform the best, with the Prefix feature achieving an F-1 score of 66.22%. Among the token context features, the token bigrams with a window of 3 provides the best configuration (almost 30% F-1). Dictionary-based features, both generic and domain-specific, have a poor performance, which is associated with their lack of discriminative power.

Figure 1 depicts the F-1 scores achieved in the one-feature setting. For an easier analysis, we’ve grouped the features according to the categories used to describe them in the previous section. It can be clearly seen from this graph that the most important feature of the model is the Prefix feature, which alone has achieved an F-1 score of 66.22% – i.e., around 5% less than the entire MALLET model. On the other hand, it seems in general that most of the simple and morphological features achieve reasonable results, in the range of 40% F-1, exceptions being the digits and punctuation features that have performed far less satisfactory. This is, however, not surprising since the elements that compose these features are not as present in the data as are the elements targeted by the other features. A particularly negative result has been the one of the Root (LEX) feature, which we have expected to perform at least as good as the Root (NLP) feature. A closer look into this aspect has revealed that the vast majority of words present in the corpus were, in reality, absent from the SPECIALIST lexicon, and consequently have been represented in the model by the same token (i.e., *@*). Good results are achieved by the most discriminant features, and hence the uniform representation of the tokens by this feature has provided a clear explanation behind the poor F-1 score. A similar behaviour (yet with a smaller difference) is observable also in the case of POS (LEX) vs. POS (NLP). Here, the difference has been provided by the wealth of representations used by the features: the SPECIALIST lexicon represents part of speech tags via a fairly concise set of tags (e.g., noun, adj, …), while an NLP framework (GATE, in our case) uses a more varied and specific set of tags (e.g., NN, NNP or NNS – all denoting nouns).

In the token context category, we’ve experimented with different configurations – the graph depicts only the best performing configurations in the unigram, bigram and trigram settings. We can see that the Token_Bi3 configuration outperforms the other two, and overall it has actually improved the final classification results, unlike the other options (or combinations of them) that have decreased the performance. Finally, the graph shows that all the dictionary features achieve poor results, although not surprisingly since they suffer from the same issue like the POS (LEX) or Root (LEX) features.

An even better view of the role of the features in classification is provided by the leave-one-out setting. Table 6 lists the achieved F-1 scores, where the Feature column presents the feature that has been left out. Here, we can clearly see the decrease in performance when the Prefix or Token_Bi3 features are left out, hence proving their importance for the classification model. It is also interesting to observe that the model without the Prefix feature performs worse than the Prefix feature alone. The rest of the features have little impact over the model providing an increase of up to 1% to the overall F-1 score.

**Table 6:** Evaluation results for MALLET ten-fold cross validation with leave-one-out feature.

Feature	P (%)	R (%)	F-1 (%)
Prefix	61.59	52.59	56.74
Root (NLP)	75.72	64.50	69.66
M_Punct	75.06	64.17	69.19
M_Vowels	76.65	64.93	70.30
Root (LEX)	72.90	62.32	67.20
Suffix	76.07	65.60	70.43
POS (LEX)	75.01	64.48	69.34
M_Digits	75.69	64.98	69.93
M_Shape	76.66	64.50	70.06
M_Bshape	75.34	65.92	70.31
POS (NLP)	75.50	65.40	70.09
Token_Bi3	65.05	49.53	56.24
D_Generic	75.72	64.57	69.70
D_Domain	76.93	65.51	70.76

This overview shows the individual importance of each of the features in the overall classification model. The large majority of features have very little impact over the model, i.e., a decrease in performance of 1–2%. The only two features that make a difference are the Prefix and the token context (Token_Bi3) that affect the overall performance with almost 15%.

The experiments presented in these sections lead to a series of major conclusions. Firstly, hybrid classification methods depend heavily on the individual performance of the underlying classifiers used for aggregation. On the other hand, such ensembles of classifiers are able to exploit the diversity and consistency among the individual elements to reach a final decision, which is usually better than the one of these single classifiers. In our case (see Table 7), two aggregation schemes – the direct set operations and the voting mechanism – have performed better than the best individual classifier, with the remark that the third one would have achieved similar results if it wouldn’t have relied also on the worst performing classifier (i.e., CRF++). Nevertheless, the efficiency and applicability of such hybrid methods requires consideration on a per use-case basis.

**Table 7:** Comparative overview of the evaluation results – with and without domain-specific dictionaries.

Veto owner	Without dictionaries			With dictionaries		
	P (%)	R (%)	F-1 (%)	P (%)	R (%)	F-1 (%)
Individual classification (Mallet)	*76.93*	65.51	70.76	*74.91*	63.99	69.02
Simple set operation	65.42	*78.33*	71.30	64.24	*78.49*	70.66
Aggregated set operation	70.05	66.97	68.48	69.76	67.53	68.62
Voting	66.14	77.84	**71.52**	65.29	77.95	**71.06**

This comparative overview shows the difference in performance between all aggregation techniques. We can see that this difference is of almost 1% between the best performing individual classifier and the best aggregation technique – the voting mechanism.

Secondly, as already noted, our experiments have showed that MALLET performs consistently, in addition to achieving excellent precision results in diverse aggregation schemes, and hence, should be always considered as foundation for any ensemble. Finally, as we have shown, the use external domain specific dictionaries has very little impact over the classification results. Furthermore, this impact can be sometimes negative, thus making the presence of such features, in practice, undesirable, while on the positive side, eliminating dependencies on domain-specific features.

### Conclusion

In this paper, we have introduced the first annotated corpus of phenotype descriptions and provided a first hybrid method for recognising such features in free text. Our hybrid method relies on an ensemble of four classifiers (two CRF and two SVM) and has used different aggregation techniques to improve the performance when compared against the one of the individual classifiers. Experimental results have showed that, without using domain-specific dictionaries the best hybrid approach can achieve an F-1 score of 71.52%, which decreases with the introduction of such dictionaries. Overall, our experiments lead to the conclusion that using an ensemble of classifiers for chunking tasks may improve the overall accuracy, however, their performance is dependent on the goal and underlying data characteristics.

For the near future, we plan to enrich the phenotype descriptions corpus with annotated case studies extracted from scientific publications. In addition, we will make the ensemble available as a REST service and as an integrated module of the upcoming DOMEO [21] v2 platform.
